# Predictive for patients with pneumonia in pediatric intensive care unit

**DOI:** 10.3389/fped.2025.1583573

**Published:** 2025-06-06

**Authors:** Mingxuan Jia, Xiyan Hu, Lin Ji, Jiawen Lin, Jialin Liu, Yong Wang

**Affiliations:** ^1^ Middlebury College, Middlebury, VT, United States; ^2^ Stanford University, Stanford, CA, United States; ^3^ Shanghai Literature Institute of Traditional Chinese Medicine, Shanghai, China; ^4^ Yangpu Hospital of Traditional Chinese Medicine, Shanghai, China; ^5^ ChengZheng Wisdom (Shanghai) Health Sciences and Technology Co., Ltd., Shanghai, China

**Keywords:** pneumonia, intensive care unit, machine learning algorithms, paediatrics, predictive models

## Abstract

**Introduction:**

Pneumonia is globally recognized as a significant disease burden, particularly among pediatric patients in intensive care units (ICU), where its etiology is complex and prognosis often poor.

**Methods:**

Data were extracted from a pediatric-specific intensive care (PIC) database, selecting 795 pediatric pneumonia patients in ICUs (2010–2018). After applying rigorous inclusion/exclusion criteria, 543 cases formed the study cohort. We analyzed patient baseline information and 70 laboratory indicators to identify 25 prognosis-associated biomarkers. For prognostic model construction, we used stepwise regression to filter 28 variables, then Spearman and Pearson correlation analyses to identify an intersection of 14 key indicators from the top 20 features. Twelve machine learning algorithms underwent parameter tuning and combination, forming 113 model combinations for survival outcome prediction.

**Results:**

The “Stepglm [both] + GBM” combination achieved the highest average accuracy (79.4%) in both training and testing sets. Twelve prognostic variables were identified: WBC Count, Glucose, Neutrophils Count, Cystatin C, Temperature (body), Sodium (Whole Blood), Cholesterol (Total), Absolute Lymphocyte Count, Urea, Lactate, and Bilirubin (Total).

**Discussion:**

These 12 variables provide a dependable basis and novel insights for prognostic evaluation, supporting clinical diagnosis, treatment, and early intervention.

## 1 Introduction

Pneumonia is a severe lung infection caused by bacteria, viruses, or other microorganisms that predominantly affect the alveoli. Among these, bacterial and viral pneumonia are the most common forms (1). Besides infectious agents, it may also be triggered by various physical and chemical factors, immune damage, allergies, and medications. The mortality rate for pneumonia patients ranges from 15.5% to 38.2% (2). A systematic analysis of data from the Global Disease Burden Database between 1990 and 2021 indicates that an estimated 344 million cases of lower respiratory infections (LRIs), primarily due to pneumonia or bronchitis, were recorded. This number has a 95% uncertainty range of 325–364 million cases. Notably, 502,000 of these fatalities (with a range of 406,000–611,000) were children under five years old, and 254,000 deaths (ranging from 197,000 to 320,000) occurred in countries with a low Socio-Demographic Index (3). Due to the complex etiological factors and diagnostic processes of pediatric pneumonia, relying solely on a single indicator cannot accurately predict outcomes, necessitating the introduction of new approaches and methods to address existing challenges.

There is substantial evidence suggesting that artificial intelligence (AI) has shown clinical utility across various realms of medical practice (4). In laboratory diagnostics, AI has been effectively utilized in tasks such as malaria diagnosis and antimicrobial resistance profiling (5, 6). Similarly, in clinical imaging analysis, AI has aided in diagnosing pulmonary tuberculosis (7, 8). Furthermore, clinical decision support tools incorporating AI have demonstrated their value in predicting sepsis, assisting with antimicrobial prescribing, and other related tasks (9, 10). Additionally, AI has played a crucial role in managing public health outbreaks, particularly in the context of the COVID-19 pandemic (11). With the burgeoning volume and complexity of biomedical data, machine learning (ML) techniques have emerged as sophisticated and popular instruments for developing predictive models of fundamental biomedical processes (12). In the realm of disease prediction using clinical data, supervised ML algorithms, such as support vector machines, naïve Bayes, and random forests, predominate (13). In biomedical applications, feedforward neural networks, convolutional neural networks, and recurrent neural networks are primarily employed (12). In clinical big data research, suitable ML algorithms can specifically address a multitude of issues from diagnosis and prognosis to treatment recommendations (14). ML holds the potential to become a reliable tool for clinical decision support. While its widespread adoption in clinical practice is apparent, efforts to validate clinically adapted ML algorithms are ongoing. By enhancing quality standards, transparency, and interpretability of ML models, acceptance thresholds can be further lowered.

In this study, addressing the challenges of prognostic assessment for pediatric pneumonia in intensive care units, we extracted, cleansed, and analyzed data from a pediatric-specific intensive care database. Among 113 machine learning algorithm combinations evaluated, the “Stepglm [both] + GBM” combination achieved the highest average accuracy of 79.4% in both training and testing sets. Overall, by integrating statistical methods with machine learning algorithms, we successfully identified twelve key indicator variables that are closely linked to the prognosis of pediatric pneumonia patients in intensive care, and established a predictive model with high accuracy. This research not only offers new insights for clinical diagnosis and treatment but also provides a reliable foundation for early warning, intervention, and improving patient survival rates.

## 2 Data and methods

### 2.1 Data sources and preprocessing

The Paediatric Intensive Care (PIC) database (http://pic.nbscn.org) is an extensive, bilingual, single-centre repository dedicated to paediatric cases, containing data on children admitted to critical care units at a major children's hospital in China (15). This de-identified database encompasses a range of information, including vital sign measurements, medication details, laboratory results, fluid balance records, diagnostic codes, hospital stay durations, survival statistics, and additional data (16). The PIC database comprises 13,499 unique hospital admissions involving 12,881 distinct paediatric patients (ages 0–18 years) who were admitted to the critical care unit between 2010 and 2018 (16). After undergoing the Collaborative Institutional Training Initiative (CITI) training and receiving the completion report from the collaborating institution, we submitted a request to the administrators and obtained authorization to use this database.

### 2.2 Inclusion and exclusion criteria

Under the purview of this study, we identified 795 individuals diagnosed with pneumonia using the ICD-10 code “J69.101”. The exclusion criteria were delineated as follows: initially, for patients with multiple admissions, only data from the first hospitalization were retained. Subsequently, entries lacking a unique identifier (SUBJECT_ID), an indicator of mortality (HOSPITAL_EXPIRE_FLAG), and “LABEVENTS” data were excluded from the data set. To ensure data integrity, we also eliminated incomplete “LABEVENTS” test records and those with missing values exceeding 20 percent. Missing values were filled in and predicted using the method of interpolation of mean, median and regression.

### 2.3 Baseline statistics

For laboratory test results, the database explicitly distinguishes between ranges of indicators (low, Z-core, high), and imputation for such missing values needs to be stratified according to these ranges. First, normality tests were conducted for each variable; continuous variables that follow a normal distribution are described using the mean ± standard deviation (17). For continuous data that do not follow a normal distribution, the median and interquartile range represent central and dispersion tendencies (18). Qualitative categorical data were described by the frequency and probability of each category (19). Differences between groups for normally distributed continuous variables were analyzed using independent sample *t*-tests to compare means (20). When the overall distribution of two sample groups differs, the rank-sum test is used to compare medians across multiple independent samples (21). Chi-square tests were used to analyze group differences in categorical data (22). All statistical analyses and inter-group difference tests were performed using R language scripts, with necessary R packages including “Hmisc”, “car”, “mice”, “openxlsx”, “dplyr”, “tidyverse”, “stats”, and “reshape2”.

### 2.4 Regression analyses

Linear regression is a widely used technique in clinical medical statistics for addressing various research questions and objectives (23). Multiple linear regression is more aligned with clinical practice, modeling the relationship between multiple independent predictors and a single outcome variable, with results dependent on the diagnostic and therapeutic research of multiple factors (24, 25). The intrinsic logic of logistic regression is to convert binary results into continuous results, i.e., the log odds or logit of the event (26). For the aforementioned analyses, R language's base package, which includes basic statistical functions, as well as packages like “caret” and “e1071”, provide advanced model training and evaluation capabilities.

### 2.5 Variable screening

In this study's stepwise regression method, the “readr” function was first used to read the data and check for missing rows. To enhance algorithm efficiency, both forward and backward stepwise regression were used iteratively. Finally, the Akaike Information Criterion (AIC), a standard for assessing the goodness of fit of statistical models, was compared; the model with the lowest AIC value was considered the optimal one.

After the initial screening, the correlation of variables with patient survival outcomes was analyzed. The Pearson correlation coefficient is suitable for linear relationships between two variables that are both continuous and normally distributed (27). The Spearman correlation coefficient uses ranks for analysis, making it applicable to a wider range of distributions compared to Pearson's coefficient (28). Due to the complexity of clinical data, we combined the results from both methods to obtain the variables for modeling.

To observe the impact of selected features on decision outcomes and their interactions, the RF algorithm was used to assess feature importance, indicating each feature's contribution to the random forest. Higher importance reflects greater influence on the forest composition and decision outcomes (29). Gray relational analysis, a multi-factor statistical method, was used to measure the degree of association between factors based on their development trends, referred to as “gray relational degree” (30). Visualization of the analysis results was accomplished using the “ggplot2” package.

### 2.6 Machine learning model construction

We selected the following twelve common ML algorithms to form a total of 113 combinations: Lasso, Stepglm, Generalized glmBoost, SVM, Ridge, Enet, Partial Least Squares Regression with Generalized Linear Model (plsRglm), RF, GBM, Linear Discriminant Analysis (LDA), XGBoost, and Naive Bayes. Most of these algorithms have been widely used in clinical predictive models, but there are three methods with relatively limited applications. PLS-R-GLM can handle both complete and incomplete datasets and is an extension of partial least squares regression for general linear models (31). LDA, a classic supervised learning algorithm, is mainly used for dimensionality reduction and classification tasks. It generalizes Fisher's linear discriminant method, aiming to identify a linear combination of features that separates two classes (32). Naive Bayes, a very simple classification method for features assumed to be independent, calculates the probability of each class given the instance to be classified and assigns it to the class with the highest probability (33).

The final modeling dataset was split into training and test sets in a 7:3 ratio, utilizing 17 R packages for data import, predictive model construction, evaluation, and result visualization, namely “openxlsx”, “seqinr”, “plyr”, “randomForestSRC”, “glmnet”, “plsRglm”, “gbm”, “caret”, “mboost”, “e1071”, “BART”, “MASS”, “snowfall”, “xgboost”, “ComplexHeatmap”, “RColorBrewer”, and “pROC”. During the actual computation process, several details need careful attention. For instance, pre-training aggregates the variable selection process for each method to reduce computation load. Setting a seed in modeling ensures reproducibility of results. After calculating the Area Under the Curve (AUC) for the training and test sets, we will compute the mean AUC for each algorithm across all cohorts and rank the algorithms by their mean AUC in descending order to determine the best-performing model.

## 3 Results

### 3.1 Baseline statistics results

**Figure 1:**
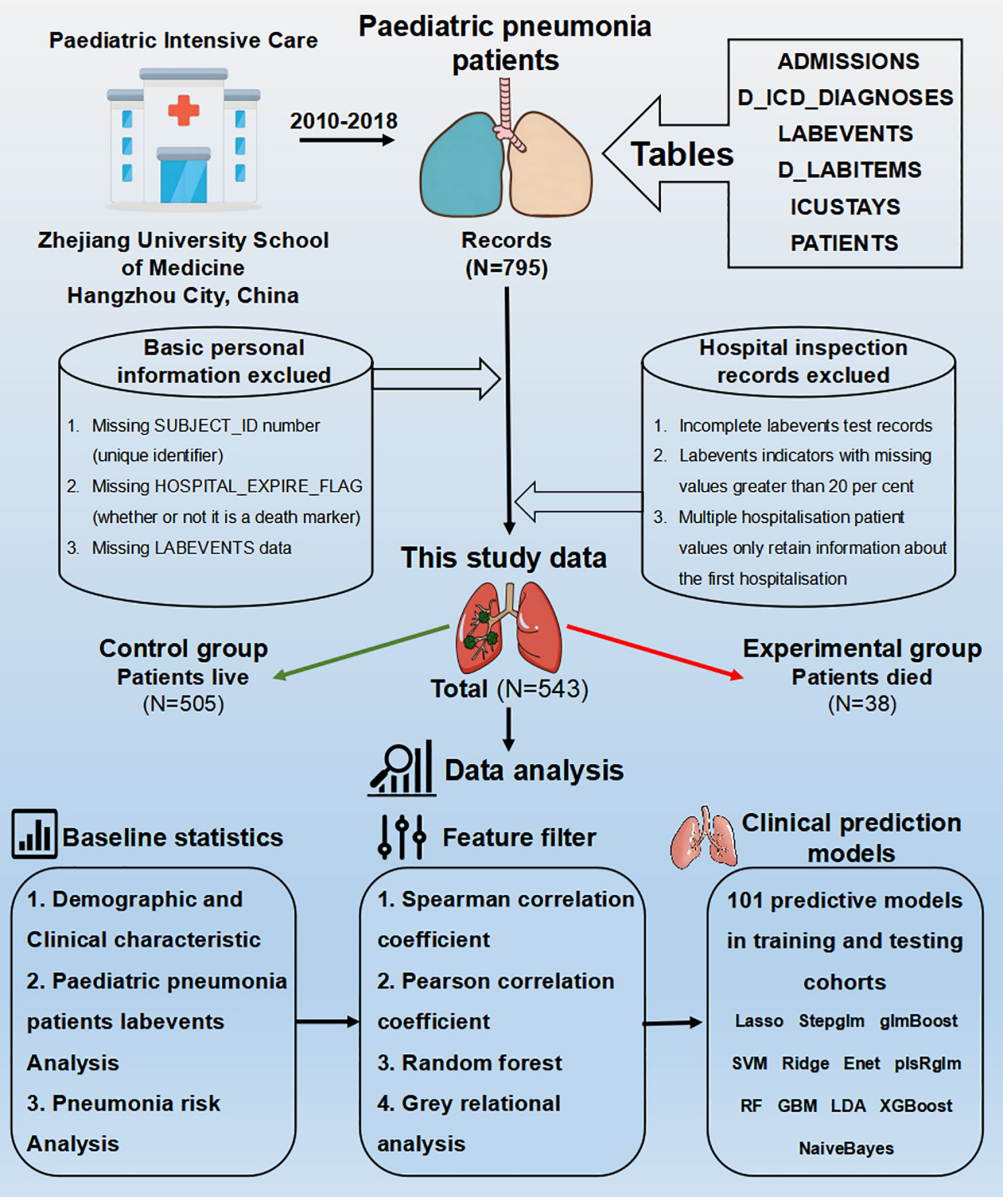
Flow diagram of this study.

The study process is illustrated in Figure 1, with a total sample size of 543, comprising 38 cases in the experimental group (deceased) and 505 cases in the control group (surviving). According to baseline statistics (Table 1), exhibited a wide age range and extended mean survival days, with no significant difference between the groups (*P* = 0.259), revealing the diversity in pneumonia progression. In both the experimental and control groups, the gender distribution of patients was similar, showing no significant difference (*P* = 0.836), thereby excluding gender as a factor in disease progression. Though ICU length of stay, temperature (body), heart rate, and respiratory rate fluctuated between the groups, there were no significant differences. However, the white blood cell (WBC) count showed a significant difference between the experimental and control groups (*P* = 0.021), potentially indicating an influential marker for disease progression and mortality in patients. Significant differences were also found in biochemical markers such as Standard Base Excess, Bicarbonate, and Alanine Aminotransferase (ALT) (*P*-values are 0.156, 0.087, and 0.002, respectively), possibly reflecting special changes in liver or acid-base balance function in some patients and associating with disease severity. Cholesterol and partial pressure of carbon dioxide (pCO2) levels, which reflect specific metabolic and physiological processes, also showed significant differences (*P* = 0.014 and 0.044). Compared to the control group, experimental group patients had higher levels of urea and Uric Acid (Urine) (*P*-values are 0.008 and 0.007), suggesting potential kidney damage or uric acid excretion issues due to pneumonia. A higher proportion of low glucose levels was observed in the experimental group (*P* = 0.001), necessitating clinical attention to hypoglycemia. Elevated lactate levels in the experimental group (*P* = 0.045) may indicate tissue hypoxia or abnormal glucose metabolism. Lactate Dehydrogenase was significantly higher in both groups, especially in the experimental group (*P* = 0.03), suggesting potential myocardial injury. Actual Base Excess reflects the body's acid-base balance. Hemoglobin (Hb) was significantly higher in the control group (*P* = 0.033), indicating better oxygen transport capacity in surviving patients. Notably, higher proportions of elevated Aspartate Aminotransferase, Bilirubin (Direct and Total) in the experimental group suggest potential liver dysfunction linked to poor prognosis. Significant differences in Lipase levels between the experimental and control groups (*P* < 0.001) may relate to pancreatic function.

**Table 1:** Demographic and clinical characteristic of paediatric pneumonia patients.

Variable type	Total (*N* = 543)		Experimental group (*N* = 38, Patients died)		Control group (*N* = 505, Patients live)		*P* value
	Number (%)	Mean ± SD	Number (%)	Mean ± SD	Number (%)	Mean ± SD	
Age (days)	–	574.16 ± 956.31	–	743.23 ± 1,437.06	–	561.44 ± 910.80	0.259
Gender	F: 223 (41%)	–	F: 15 (39%)	–	F: 208 (41%)	–	0.836
	M: 320 (59%)	–	M: 23 (61%)	–	M: 297 (59%)	–	
ICU stays days	–	11.55 ± 18.92	–	14.41 ± 15.58	–	11.33 ± 19.15	0.334
Temperature (℃)	–	37.1 ± 0.814	–	37.29 ± 0.839	–	37.09 ± 0.811	0.145
Heart rate (bpm)	–	135.06 ± 24.68	–	129.16 ± 28.80	–	135.51 ± 24.32	0.193
Respiratory rate (bpm)	–	49.96 ± 44.14	–	58.08 ± 62.15	–	49.32 ± 42.50	0.24
Diastolic pressure (mmHg)	–	57.05 ± 21.32	–	55.66 ± 9.16	–	57.15 ± 21.96	0.677
Systolic pressure (mmHg)	–	96.14 ± 15.21	–	95.61 ± 11.80	–	96.18 ± 15.44	0.778
WBC count (×10^9^/L)	–	11 ± 7.71	–	13.78 ± 11.61	–	10.79 ± 7.31	**0.021**
Low	60 (11.1%)	–	4 (10.5%)	–	56 (11.1%)	–	0.479
*Z*-score	329 (60.6%)	–	20 (52.6%)	–	309 (61.2%)	–	
High	154 (28.3%)	–	14 (36.7%)	–	140 (27.7%)	–	
Standard base excess (mmol/L)	–	0.37 ± 5.02	–	−0.89 ± 5.66	–	0.47 ± 4.96	0.156
Low	114 (21%)	–	15 (39.5%)	–	99 (19.6%)	–	**0.012**
*Z*-score	292 (53.8%)	–	14 (36.8%)	–	278 (55%)	–	
High	137 (25.2%)	–	9 (23.7%)	–	128 (25.4%)	–	
Bicarbonate	–	24.57 ± 4.15	–	23.46 ± 4.88	–	24.65 ± 4.09	0.087
Low	88 (16.2%)	–	13 (34.2%)	–	75 (14.9%)	–	**0.005**
*Z*-score	329 (60.6%)	–	16 (42.1%)	–	313 (62%)	–	
High	126 (23.2%)	–	9 (23.7%)	–	117 (23.1%)	–	
Alanine aminotransferase (U/L)	–	61.55 ± 197.35	–	156.42 ± 519.33	–	54.41 ± 146.13	**0.002**
*Z*-score	434 (79.9%)	–	29 (76.3%)	–	405 (80.2%)	–	0.564
High	109 (20.1%)	–	9 (23.7%)	–	100 (19.8%)	–	
Monocytes (%)	–	7.57 ± 4.63	–	5.98 ± 2.50	–	7.69 ± 4.73	**<0.001**
Low	100 (18.4%)	–	7 (18.4%)	–	93 (18.4%)	–	0.494
*Z*-score	425 (78.3%)	–	31 (81.6%)	–	394 (78%)	–	
High	18 (3.3%)	–	0 (0%)	–	18 (3.6%)	–	
Cholesterol, total (mmol/L)	–	3.21 ± 1.02	–	3.35 ± 1.43	–	3.2 ± 0.978	0.523
Low	221 (21%)	–	16 (39.5%)	–	205 (19.6%)	–	**0.014**
*Z*-score	312 (53.8%)	–	19 (36.8%)	–	293 (55%)	–	
High	10 (25.2%)	–	3 (23.7%)	–	7 (25.4%)	–	
pCO2 (mmHg)	–	46.11 ± 15.82	–	51.09 ± 19.57	–	45.74 ± 15.46	**0.044**
Low	92 (16.9%)	–	7 (18.4%)	–	85 (16.8%)	–	0.185
*Z*-score	260 (47.9%)	–	13 (34.2%)	–	247 (48.9%)	–	
High	191 (35.2%)	–	18 (47.4%)	–	173 (34.3%)	–	
Methemoglobin (%)	–	0.79 ± 1.15	–	0.61 ± 0.31	–	0.8 ± 1.19	**0.013**
*Z*-score	542 (99.8%)	–	38 (100%)	–	504 (99.8%)	–	0.784
High	1 (0.2%)	–	0 (0%)	–	1 (0.2%)	–	
RDW (%)	–	14.78 ± 2.067	–	15.22 ± 1.88	–	14.74 ± 2.08	**0.033**
*Z*-score	506 (93.2%)	–	31 (81.6%)	–	469 (92.9%)	–	0.289
High	37 (6.8%)	–	7 (18.4%)	–	36 (7.1%)	–	
Creatinine (μmol/L)	–	44.6 ± 23.79	–	52.87 ± 53.41	––	43.98 ± 19.84	**0.026**
Low	8 (1.4%)	–	1 (2.6%)	–	7 (1.4%)	–	0.827
*Z*-score	520 (95.8%)	–	36 (94.8%)	–	484 (95.8%)	–	
High	15 (2.8%)	–	1 (2.6%)	–	14 (2.8%)	–	
Bilirubin, indirect (μmol/L)	–	14.61 ± 29.84	–	20.06 ± 42.02	–	14.2 ± 28.73	**0.027**
Low	10 (1.8%)	–	0 (0%)	–	10 (2%)	–	0.227
*Z*-score	451 (83.1%)	–	29 (76.3%)	–	422 (83.6%)	–	
High	82 (15.1%)	–	9 (23.7%)	–	73 (14.4%)	–	
Urea (mmol/L)	–	3.74 ± 2.61	–	4.83 ± 3.91	–	3.66 ± 2.47	**0.008**
Low	84 (15.5%)	–	5 (13.2%)	–	79 (15.7%)	–	0.118
*Z*-score	419 (77.2%)	–	27 (71.1%)	–	392 (77.6%)	–	
High	40 (7.3%)	–	6 (15.7%)	–	34 (6.7%)	–	
Uric acid, urine (μmol/L)	–	263.35 ± 140.74	–	334.61 ± 198.28	–	257.99 ± 134.18	**0.007**
Low	95 (17.5%)	–	3 (7.9%)	–	92 (18.2%)	–	**0.039**
*Z*-score	361 (66.5%)	–	24 (63.2%)	–	337 (66.7%)	–	
Glucose (mmol/L)	–	6.99 ± 3.40	–	6.65 ± 2.41	–	7.03 ± 3.46	0.412
Low	12 (2.2%)	–	4 (10.5%)	–	8 (1.6%)	–	**0.001**
*Z*-score	248 (45.7%)	–	14 (36.9%)	–	234 (46.3%)	–	
High	283 (52.1%)	–	20 (52.6%)	–	263 (52.1%)	–	
Lactate (mmol/L)	–	2.37 ± 1.64	–	2.88 ± 2.24	–	2.33 ± 1.58	**0.045**
Low	2 (0.3%)	–	0 (0%)	–	2 (0.4%)	–	0.918
*Z*-score	205 (37.8%)	–	14 (36.8%)	–	191 (37.8%)	–	
High	336 (61.9%)	–	24 (63.2%)	–	312 (61.8%)	–	
Lactate dehydrogenase (U/L)	–	542.99 ± 822.99	–	747.21 ± 1,412.79	–	527.62 ± 760.57	0.349
Low	1 (0.2%)	–	0 (0%)	–	1 (0.2%)	–	**0.030**
*Z*-score	254 (46.8%)	–	10 (26.3%)	–	244 (48.3%)	–	
High	288 (53%)	–	28 (73.7%)	–	260 (51.5%)	–	
Actual base excess (mmol/L)	–	0.05 ± 4.75	–	0.11 ± 4.69	–	﹣0.83 ± 5.47	0.240
Low	117 (21.5%)	–	14 (36.8%)	–	103 (20.4%)	–	**0.042**
*Z*-score	302 (55.6%)	–	15 (39.5%)	–	287 (56.8%)	–	
High	124 (22.8%)	–	9 (23.7%)	–	115 (22.8%)	–	
Eosinophil (%)	–	1.31 ± 1.94	–	0.89 ± 1.25	–	1.34 ± 1.98	**0.050**
Low	338 (62.2%)	–	26 (68.4%)	–	312 (61.7%)	–	0.414
*Z*-score	186 (34.3%)	–	12 (31.6%)	–	174 (34.5%)	–	
High	19 (3.5%)	–	0 (0%)	–	19 (3.8%)	–	
pH	–	7.37 ± 0.09	–	7.33 ± 0.13	–	7.37 ± 0.08	**0.031**
Low	195 (35.9%)	–	21 (55.3%)	–	174 (34.4%)	–	**0.027**
*Z*-score	276 (50.8%)	–	12 (31.6%)	–	264 (52.3%)	–	
High	72 (13.3%)	–	5 (13.1%)	–	67 (13.3%)	–	
Calculated bicarbonate, whole blood (mmol/L)	–	25.15 ± 5.29	–	25.24 ± 5.68	–	25.14 ± 5.26	0.91
Low	102 (18.8%)	–	10 (26.4%)	–	92 (18.2%)	–	**0.039**
*Z*-score	307 (56.5%)	–	14 (36.8%)	–	293 (58%)	–	
High	134 (24.7%)	–	14 (36.8%)	–	120 (23.8%)	–	
Asparate aminotransferase (U/L)	–	117.16 ± 545.01	–	98.49 ± 356.10	–	365.24 ± 1,598.75	**0.004**
Low	6 (1.1%)	–	0 (0%)	–	6 (1.2%)	–	0.794
*Z*-score	365 (67.2%)	–	26 (68.4%)	–	339 (97.1%)	–	
High	172 (31.7%)	–	12 (31.6%)	–	160 (31.7%)	–	
Hemoglobin Hb (g/dl)	–	94.27 ± 40.14	–	80.91 ± 48.47	–	95.28 ± 39.32	**0.033**
Low	446 (82.1%)	–	30 (78.9%)	–	416 (82.4%)	–	0.613
*Z*-score	91 (16.8%)	–	7 (18.5%)	–	84 (16.6%)	–	
High	6 (1.1%)	–	1 (2.6%)	–	5 (1%)	–	
Lipase	–	0.52 ± 3.09	–	–	–	0.56 ± 3.21	**<0.001**
*Z*-score	543 (100%)	–	38 (100%)	–	505 (100%)	–	
Bilirubin, direct (μmol/L)	–	5.27 ± 9.96	–	7.22 ± 6.74	–	5.12 ± 10.15	**0.009**
*Z*-score	419 (77.2%)	–	21 (55.3%)	–	398 (78.8%)	–	**0.001**
High	124 (22.8%)	–	17 (44.7%)	–	107 (21.2%)	–	
Bilirubin, total (μmol/L)	–	19.87 ± 36.41	–	27.22 ± 45.37	–	19.32 ± 35.64	**0.01**
Low	114 (20%)	–	5 (13.2%)	–	109 (21.6%)	–	**0.039**
*Z*-score	336 (61.9%)	–	21 (55.3%)	–	315 (62.4%)	–	
High	93 (17.1%)	–	12 (31.5%)	–	81 (16.0%)	–	

Bold values indicate statistical significance (*p* < 0.05).

### 3.2 Linear regression model

The linear regression analysis investigated the impact of various biomarkers and clinical parameters on the survival of pediatric pneumonia patients in the pediatric intensive care unit (PPICU). The results, as shown in Table 2, reveal that the regression coefficient for WBC count is 0.003, with a t-value of 2.317 and a *P*-value of 0.021, indicating a significant association between high white blood cell counts and the survival chances of patients, underscoring its importance in clinical monitoring. Similarly, the regression coefficient for ALT is 0.000, with a t-value of 3.097 and a *P*-value of 0.002, suggesting that elevated ALT levels may reflect liver damage or disease severity, highlighting the necessity for physicians to monitor liver function. The monocyte percentage regression coefficient is −0.005, with a t-value of −2.204 and a *P*-value of 0.028, demonstrating its association with reduced survival rates, further supporting the role of inflammatory response in patient outcomes. The mean pCO2 regression coefficient is 0.001, with a t-value of 2.019 and a *P*-value of 0.044, indicating that higher pCO2 levels might reflect respiratory insufficiency, warranting close attention. The regression coefficients for creatinine and urea are 0.001 (*P* = 0.026) and 0.011 (*P* = 0.008), respectively, pointing to a correlation between renal function parameters and survival status, emphasizing the necessity of monitoring renal function in patients. Additionally, the lactate regression coefficient is 0.13 (*P* = 0.045), indicating elevated levels often signify tissue hypoxia or severe infection, necessitating attention to the metabolic status of patients. Intriguingly, the pH value regression coefficient is −0.402 (*P* = 0.001), showing a significant impact on the survival prognosis of patients, suggesting that acid-base imbalance could lead to severe consequences.

**Table 2:** General linear regression analysis results.

Patients died or live	Regression coefficient	*t*	R2	*P*-value	95% CI
Constant	0.034	1.783	–	–	–
WBC count (×10^9^/L)	0.003	2.317	0.010	**0.021**	(0.000, 0.006)
Constant	0.059	5.221	–	–	–
Alanine aminotransferase (U/L)	0.000	3.097	0.016	**0.002**	(0.000, 0.000)
Constant	0.109	5.223	–	–	–
Monocytes (%)	−0.005	−2.204	0.009	**0.028**	(−0.010, −0.001)
Constant	0.006	0.167	–	–	–
pCO2 (mmHg)	0.001	2.019	0.007	**0.044**	(0.000,0.003)
Constant	0.024	1.046	–	–	–
Creatinine (μmol/L)	0.001	2.231	0.009	**0.026**	(0.000, 0.002)
Constant	0.028	1.463	–	–	–
Urea (mmol/L)	0.011	2.684	0.013	**0.008**	(0.003, 0.019)
Constant	0.004	0.155	–	–	–
Uric acid, urine (μmol/L)	0.000	3.265	0.019	**0.001**	(0.000, 0.000)
Constant	0.038	1.984	–	–	–
Lactate (mmol/L)	0.13	2.005	0.007	**0.045**	(0.000, 0.027)
Constant	3.029	3.295	–	–	–
pH	−0.402	−3.219	0.019	**0.001**	(−0.647, −0.157)
Constant	0.063	5.671	–	–	–
Asparate aminotransferase (U/L)	5.855E-5	2.930	0.016	**0.004**	(0.000, 0.000)
Constant	0.125	4.473	–	–	–
Hemoglobin Hb (g/dl)	−0.001	−2.135	0.008	**0.033**	(−0.001, 0.000)

Bold values indicate statistical significance (*p* < 0.05).

### 3.3 Logistic regression model

Regarding the binary logistic regression analysis of the first categorical variable as the reference class, the results (Table 3) indicate that actual base excess and standard base excess have significant predictive effects on whether patients survive or succumb. Specifically, the low and high levels of these variables both exhibit statistical differences with *P*-values less than 0.05. This further highlights the importance of these testing indicators at certain excessive levels, increasing the mortality risk of patients, making them crucial variables for focus and intervention in patient clinical treatment. Furthermore, elevated levels of cholesterol, total and uric acid, urine also significantly affect the prediction of patient outcomes (*P*-values of 0.021 and 0.026, respectively). High cholesterol and uric acid levels might indicate a higher disease severity in patients, potentially correlating with the patient's prognosis. Additionally, lactate dehydrogenase shows significant relevance in predicting patient outcomes. This could be because changes in lactate dehydrogenase levels reflect the progression of the disease or organ function status in patients. Given its high statistical significance, this variable might warrant further study. Finally, elevated levels of direct bilirubin and total bilirubin are found to have a significant correlation with patient outcomes. This suggests that bilirubin levels could be crucial indicators in assessing pediatric pneumonia patients' critical care process. Higher bilirubin levels might indicate liver function impairment or the presence of other complications.

**Table 3:** Logistic regression results (reference category to first).

Patients died or live	B	S.E.	Wald	df	*P*-value	Exp(B)	95% CI (Upper limit)	95% CI (lower limit)
Standard base excess (mmol/L)								
Low			8.276	2	**0.016**			
*Z*-score	−1.101	0.39	7.993	1	**0.005**	0.332	0.155	0.713
High	−0.768	0.442	3.012	1	0.083	0.464	0.195	1.104
Constant	−1.887	0.277	46.387	1	0	0.152		
Bicarbonate								
Low			9.632	2	**0.008**			
*Z*-score	−1.221	0.395	9.561	1	**0.002**	0.295	0.136	0.639
High	−0.812	0.458	3.144	1	0.076	0.444	0.181	1.089
Constant	−1.753	0.3	34.03	1	0	0.173		
Cholesterol, total (mmol/L)								
Low			6.702	2	0.035			
*Z*-score	−0.185	0.351	0.278	1	0.598	0.831	0.417	1.654
High	1.703	0.737	5.336	1	**0.021**	5.491	1.294	23.293
Constant	−2.55	0.26	96.54	1	0	0.078		
Uric acid, urine (μmol/L)								
Low			6.014	2	**0.049**			
*Z*-score	0.781	0.624	1.569	1	0.21	2.184	0.643	7.414
High	1.49	0.67	4.955	1	**0.026**	4.439	1.195	16.487
Constant	−3.423	0.587	34.044	1	0	0.033		
Glucose (mmol/L)								
Low			10.118	2	**0.006**			
*Z*-score	−2.123	0.671	10.001	1	**0.002**	0.12	0.032	0.446
High	−1.883	0.655	8.271	1	**0.004**	0.152	0.042	0.549
Constant	−0.693	0.612	1.281	1	0.258	0.5		
Lactate dehydrogenase (U/L)								
Low			6.497	2	**0.039**			
*Z*-score	18.008	40,192.53	0	1	1	66,208,218.8	0	.
High	18.974	40,192.53	0	1	1	173,974,827.2	0	.
Constant	−21.203	40,192.53	0	1	1	0		
Actual base excess (mmol/L)								
Low			6.048	2	0.049			
*Z*-score	−0.956	0.389	6.038	1	0.014	0.385	0.179	0.824
High	−0.552	0.448	1.517	1	0.218	0.576	0.239	1.386
Constant	−1.996	0.285	49.086	1	0	0.136		
pH								
Low			6.836	2	**0.033**			
*Z*-score	−0.977	0.375	6.788	1	**0.009**	0.377	0.181	0.785
High	−0.481	0.518	0.861	1	0.353	0.618	0.224	1.707
Constant	−2.115	0.231	83.784	1	0	0.121		
Calculated bicarbonate, whole blood (mmol/L)								
Low			6.171	2	**0.046**			
*Z*-score	−0.822	0.431	3.638	1	0.056	0.44	0.189	1.023
High	0.071	0.437	0.026	1	0.871	1.073	0.456	2.526
Constant	−2.219	0.333	44.42	1	0	0.109		
Bilirubin, direct (μmol/L)								
*Z*-score								
High	1.102	0.344	10.271	1	**0.001**	3.011	1.534	5.909
Constant	−2.942	0.224	172.645	1	0	0.053		
Bilirubin, total (μmol/L)								
Low			6.101	2	**0.047**			
*Z*-score	0.374	0.51	0.538	1	0.463	1.453	0.535	3.948
High	1.172	0.552	4.509	1	0.034	3.23	1.094	9.531
Constant	−3.082	0.457	45.408	1	0	0.046		

Bold values indicate statistical significance (*p* < 0.05).

Using the last categorical variable as a reference category, the results of the logistic regression analysis (Table 4) indicated that certain levels of specific biomarkers, such as elevated Cholesterol, Total, elevated Glucose, and elevated Bilirubin, Direct, were significantly associated with adverse clinical outcomes in patients (*P*-value less than 0.05). Particularly, high levels of Total Cholesterol and Glucose demonstrated substantial statistical significance in the associated regression model. Specifically, elevated levels of these biomarkers play a significant role in predicting adverse clinical outcomes in patients. These findings suggest that in clinical practice, pediatric pneumonia patients with these elevated biomarkers require closer monitoring and potentially more aggressive therapeutic interventions.

**Table 4:** Logistic regression results (reference category to last).

Patients died or live	B	S.E.	Wald	df	*P*-value	Exp(B)	95% CI (Upper limit)	95% CI (lower limit)
Standard base excess (mmol/L)								
High			8.276	2	**0.016**			
*Z*-score	0.768	0.442	3.012	1	0.083	2.155	0.905	5.128
Low	−0.334	0.44	0.574	1	0.449	0.716	0.302	1.698
Constant	−2.655	0.345	59.265	1	0	0.07		
Bicarbonate								
High			9.632	2	**0.008**			
*Z*-score	0.812	0.458	3.144	1	0.076	2.253	0.918	5.531
Low	−0.409	0.431	0.901	1	0.343	0.665	0.286	1.545
Constant	−2.565	0.346	54.981	1	0	0.077		
Cholesterol, total (mmol/L)								
High			6.702	2	**0.035**			
*Z*-score	−1.703	0.737	5.336	1	**0.021**	0.182	0.043	0.773
Low	−1.888	0.73	6.7	1	**0.01**	0.151	0.036	0.632
Constant	−0.847	0.69	1.508	1	0.22	0.429		
Uric acid, urine (μmol/L)								
High			6.014	2	0.049			
*Z*-score	−1.49	0.67	4.955	1	**0.026**	0.225	0.061	0.837
Low	−0.709	0.386	3.382	1	0.066	0.492	0.231	1.048
Constant	−1.933	0.323	35.899	1	0	0.145		
Glucose (mmol/L)								
High			10.118	2	**0.006**			
*Z*-score	1.883	0.655	8.271	1	**0.004**	6.575	1.822	23.729
Low	−0.24	0.36	0.444	1	0.505	0.787	0.389	1.593
Constant	−2.576	0.232	123.377	1	0	0.076		
Lactate dehydrogenase (U/L)								
High			6.497	2	**0.039**			
*Z*-score	−18.974	40,192.97	0	1	1	0	0	.
Low	−0.966	0.379	6.497	1	**0.011**	0.381	0.181	0.8
Constant	−2.228	0.199	125.532	1	0	0.108		
Actual base excess (mmol/L)								
High			6.048	2	**0.049**			
*Z*-score	0.552	0.448	1.517	1	0.218	1.737	0.721	4.181
Low	−0.404	0.436	0.858	1	0.354	0.668	0.284	1.569
Constant	−2.548	0.346	54.177	1	0	0.078		
pH								
High			6.836	2	**0.033**			
*Z*-score	0.481	0.518	0.861	1	0.353	1.617	0.586	4.463
Low	−0.496	0.55	0.814	1	0.367	0.609	0.207	1.789
Constant	−2.595	0.464	31.338	1	0	0.075		
Calculated bicarbonate, whole blood (mmol/L)								
High			6.171	2	**0.046**			
*Z*-score	−0.071	0.437	0.026	1	0.871	0.932	0.396	2.192
Low	−0.893	0.393	5.154	1	0.023	0.41	0.19	0.885
Constant	−2.148	0.282	57.869	1	0	0.117		
Bilirubin, direct (μmol/L)								
High								
*Z*-score	−1.102	0.344	10.271	1	**0.001**	0.332	0.169	0.652
Constant	−1.84	0.261	49.644	1	0	0.159		
Bilirubin, total (μmol/L)								
High			6.101	2	**0.047**			
*Z*-score	−1.172	0.552	4.509	1	**0.034**	0.31	0.105	0.914
Low	−0.799	0.383	4.353	1	**0.037**	0.45	0.213	0.953
Constant	−1.91	0.309	38.11	1	0	0.148		

Bold values indicate statistical significance (*p* < 0.05).

### 3.4 Prognostic variable screening

**Figure 2:**
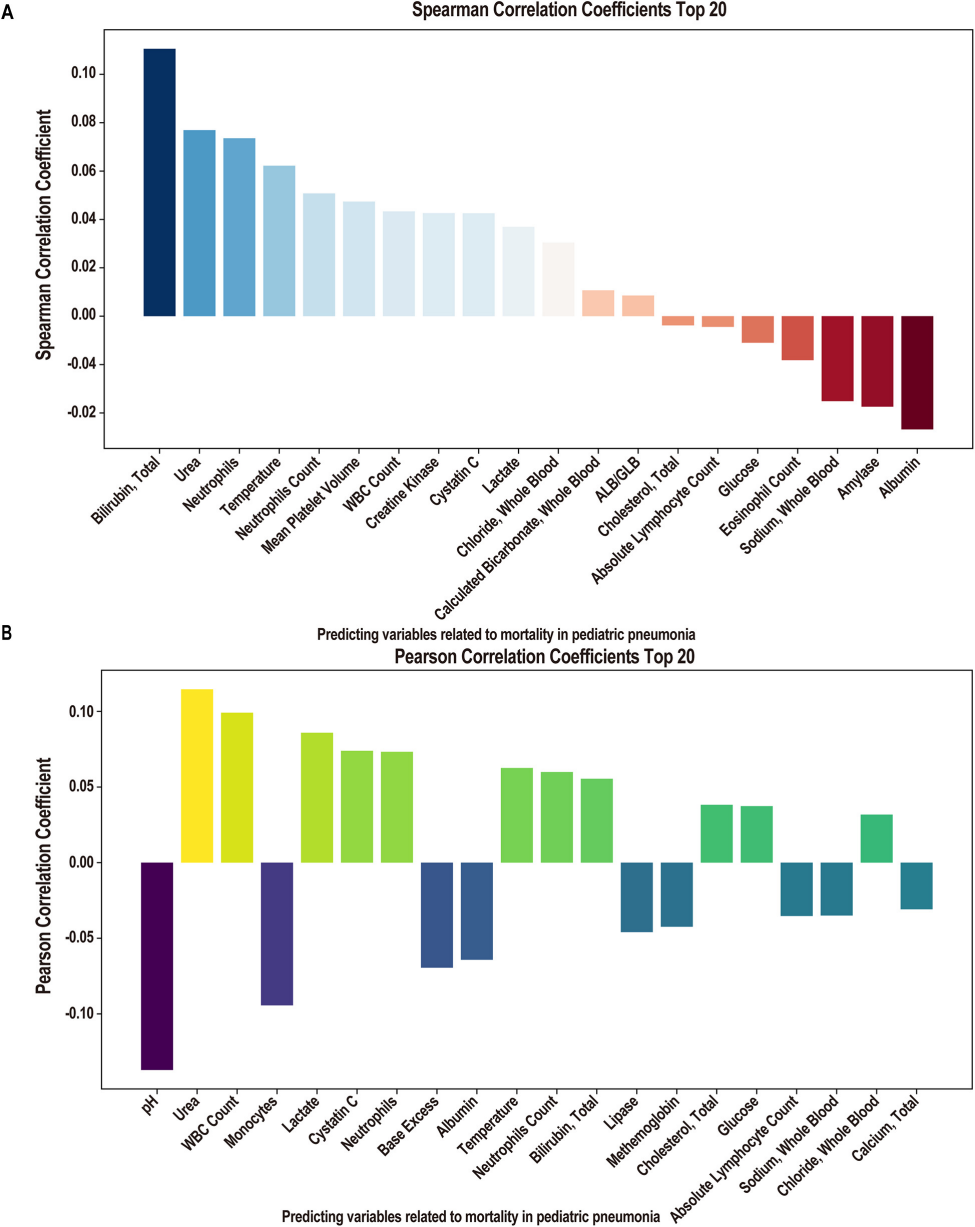
Correlation analysis of clinical factors. **(A)** Spearman correlation coefficient of importance clinical factors (Top 20 features). **(B)** Pearson correlation coefficient of importance clinical factors (Top 20 features).

**Figure 3:**
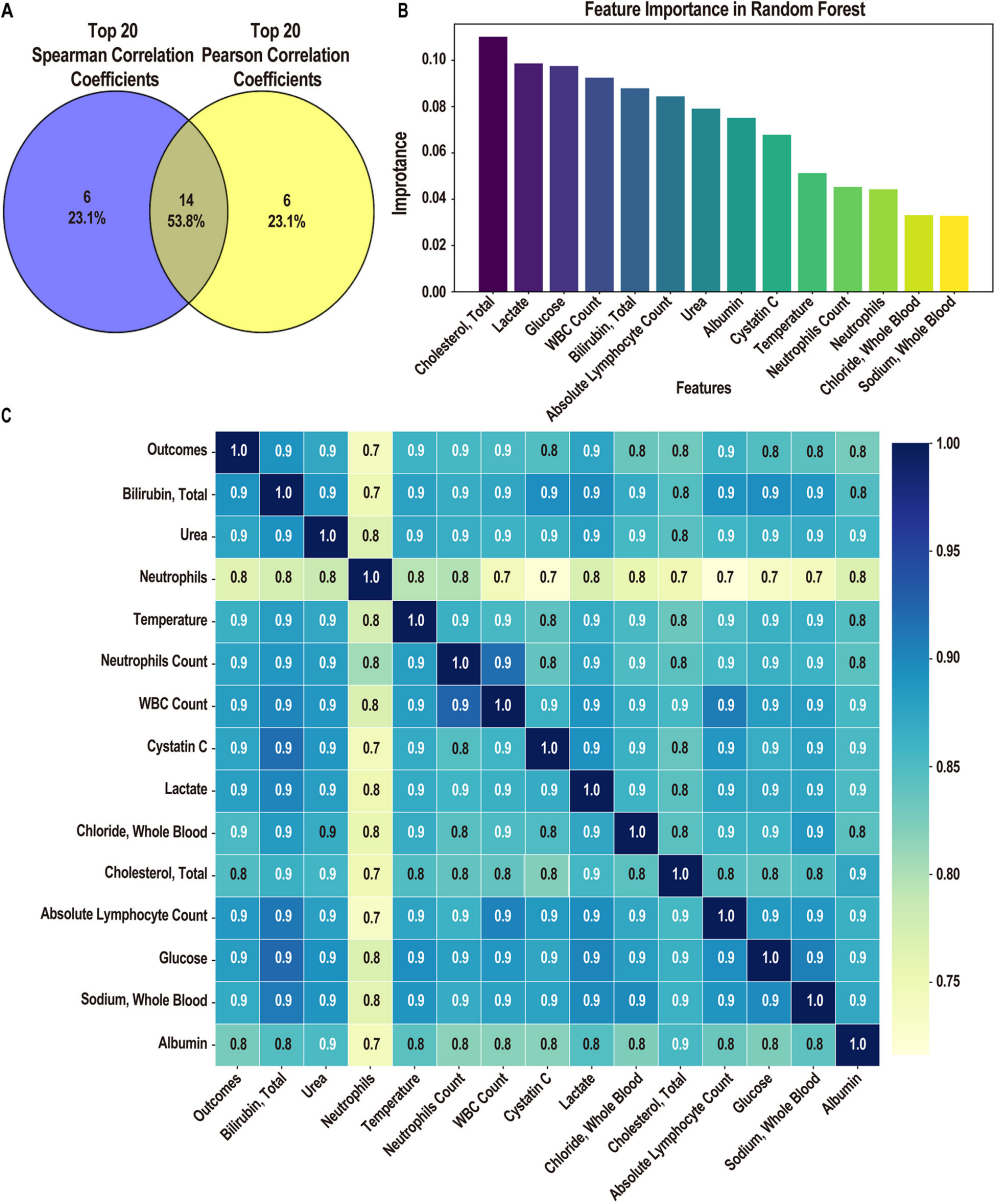
Screening of elements for modelling. **(A)** The Venn plot of spearman and Pearson correlation coefficient Top 20 lements of choice (14 features). **(B)** Fourteen features importance in fandom forest. **(C)** Grey relational analysis of importance clinical factors (14 features).

Following linear and logistic regressions on the data set, we envisioned employing ML algorithms to construct a clinical prediction model. Initially, for the mortality or survival outcomes of pediatric pneumonia patients in the PPICU, we performed stepwise regression to select a series of clinical variables. From 78 initial variables, 28 were selected for subsequent studies, with the AIC value at 228.84 and deviance at 170.84 (Table 5). In the Spearman correlation analysis's top 20 (Figure 2A), Bilirubin, Total, Urea, and Neutrophils were the three most positively correlated factors, while Albumin, Amylase, and Sodium, Whole Blood. The pH showed the strongest negative correlations in the Pearson correlation analysis (Figure 2B). Taking the intersection of the top 20 variables from both analyses (Figure 3A), we obtained 14 variables [Bilirubin, Urea, Neutrophils, Temperature (body), Neutrophils Count, WBC Count, Cystatin C, Lactate, Chloride, Cholesterol, Absolute Lymphocyte Count, Glucose, Sodium, and Albumin] to construct the ML algorithm model.

**Table 5:** Screening variable results by stepwise regression.

Correlation	Features	Df	Deviance	AIC
	Outcome∼Temperature + Albumin + ALB/GLB + WBC Count + Base Excess + Monocytes + Cholesterol, Total + Amylase + Calcium, Total + Methemoglobin + Cystatin C + Creatine Kinase + Absolute Lymphocyte Count + Chloride, Whole Blood + Sodium, Whole Blood + Urea + Mean Platelet Volume + Glucose + Lactate + Eosinophil Count + pH + Calculated Bicarbonate, Whole Blood + Serum hemolytic index + Lipase + Neutrophils + Neutrophils Count + Bilirubin, Total + Total Bile Acid	–	**170.84**	**228.84**
−	pH	1	172.99	228.99
+	Mean haemoglobin concentration	1	169.1	229.1
−	Total bile acid	1	173.21	229.21
+	ICU stays days	1	169.27	229.27
−	ALB/GLB	1	173.28	229.28
−	Serum hemolytic index	1	173.51	229.51
+	Bilirubin, indirect	1	169.52	229.52
+	Bilirubin, direct	1	169.61	229.61
+	Basophils count	1	169.62	229.62
+	Hematocrit	1	169.64	229.64
−	Absolute lymphocyte count	1	173.71	229.71
+	Alkaline phosphatase	1	169.73	229.73
+	C-reactive protein	1	169.84	229.84
+	Calcium, total	1	169.9	229.9
+	Hemoglobin	1	170.04	230.04
+	Gamma glutamyltransferase	1	170.07	230.07
+	MCV	1	170.07	230.07
−	Cystatin C	1	174.11	230.11
+	pO2	1	170.15	230.15
+	Alanine aminotransferase	1	170.28	230.28
+	Hemoglobin	1	170.29	230.29
+	Cholinesterase	1	170.32	230.32
+	Oxygen saturation	1	170.39	230.39
+	Prealbumin	1	170.39	230.39
+	Serum icteric index	1	170.40	230.40
−	Eosinophil count	1	174.42	230.42
+	Platelet count	1	170.44	230.44
+	Heart rate	1	170.45	230.45
+	Creatine kinase, MB isoenzyme	1	170.47	230.47
+	Diastolic pressure	1	170.48	230.48
+	Protein, total	1	170.49	230.49
+	Uric acid, urine	1	170.57	230.57
+	Basophils	1	170.59	230.59
+	Adenosine deaminase	1	170.61	230.61
+	Asparate aminotransferase	1	170.63	230.63
+	Phosphate	1	170.63	230.63
+	Carboxyhemoglobin	1	170.64	230.64
+	RDW	1	170.65	230.65
+	PCT	1	170.72	230.72
+	Globulin	1	170.72	230.72
+	Respiratory rate	1	170.72	230.72
+	Eosinophil	1	170.72	230.72
+	pCO2	1	170.74	230.74
+	Age (days)	1	170.77	230.77
+	Bicarbonate	1	170.80	230.80
+	Platelet distribution width	1	170.8	230.8
+	Monocyte count	1	170.81	230.81
+	Lactate dehydrogenase	1	170.81	230.81
+	Hematocrit	1	170.82	230.82
+	Systolic pressure	1	170.82	230.82
+	Creatinine	1	170.83	230.83
+	MCH	1	170.84	230.84
+	Gender	1	170.84	230.84
+	Triglycerides	1	170.84	230.84
+	Potassium	1	170.84	230.84
+	Lymphocytes, percent	1	170.84	230.84
+	Base excess	1	170.84	230.84
−	Creatine kinase	1	175.25	231.25
−	Amylase	1	175.37	231.37
−	Neutrophils	1	175.66	231.66
−	Lactate	1	175.72	231.72
−	Monocytes	1	176.13	232.13
−	Calcium, total	1	176.54	232.54
−	Bilirubin, total	1	176.57	232.57
−	Lipase	1	177.16	233.16
−	Glucose	1	177.82	233.82
−	Mean platelet volume	1	178.24	234.24
−	Base excess	1	179.24	235.24
−	Urea	1	179.41	235.41
−	Albumin	1	179.87	235.87
−	Cholesterol, total	1	180.70	236.70
−	Temperature	1	181.9	237.9
−	WBC count	1	183.28	239.28
−	Chloride, whole blood	1	184.7	240.7
−	Neutrophils count	1	184.94	240.94
−	Calculated bicarbonate, whole blood	1	185.16	241.16
−	Sodium, whole blood	1	185.39	241.39
−	Methemoglobin	1	186.84	242.84

Bold values indicate statistical significance (*p* < 0.05).

Using the RF algorithm to analyze the contribution ratios of 14 variables to the survival outcomes of patients (Figure 3B), Cholesterol, Total, Lactate, and Glucose were the three with the highest contributions. Specifically, Cholesterol levels may be closely related to the patient's nutritional status and inflammatory response. High cholesterol levels may indicate metabolic disorders, affecting the overall health assessment of patients. Lactate is an indicator of metabolic acidosis, commonly seen in hypoxia or circulatory failure, and elevated lactate levels are typically associated with poorer survival outcomes. Lastly, abnormal Glucose levels (especially hyperglycemia) are often associated with stress and infection and may reflect the patient's energy metabolism status. From the grey correlation degree analysis results, these 14 variables all exhibited strong correlations, with association coefficients ranging from 0.7 to 0.9 (Figure 3C).

### 3.5 113 machine learning algorithms to construct prognostic models

**Figure 4:**
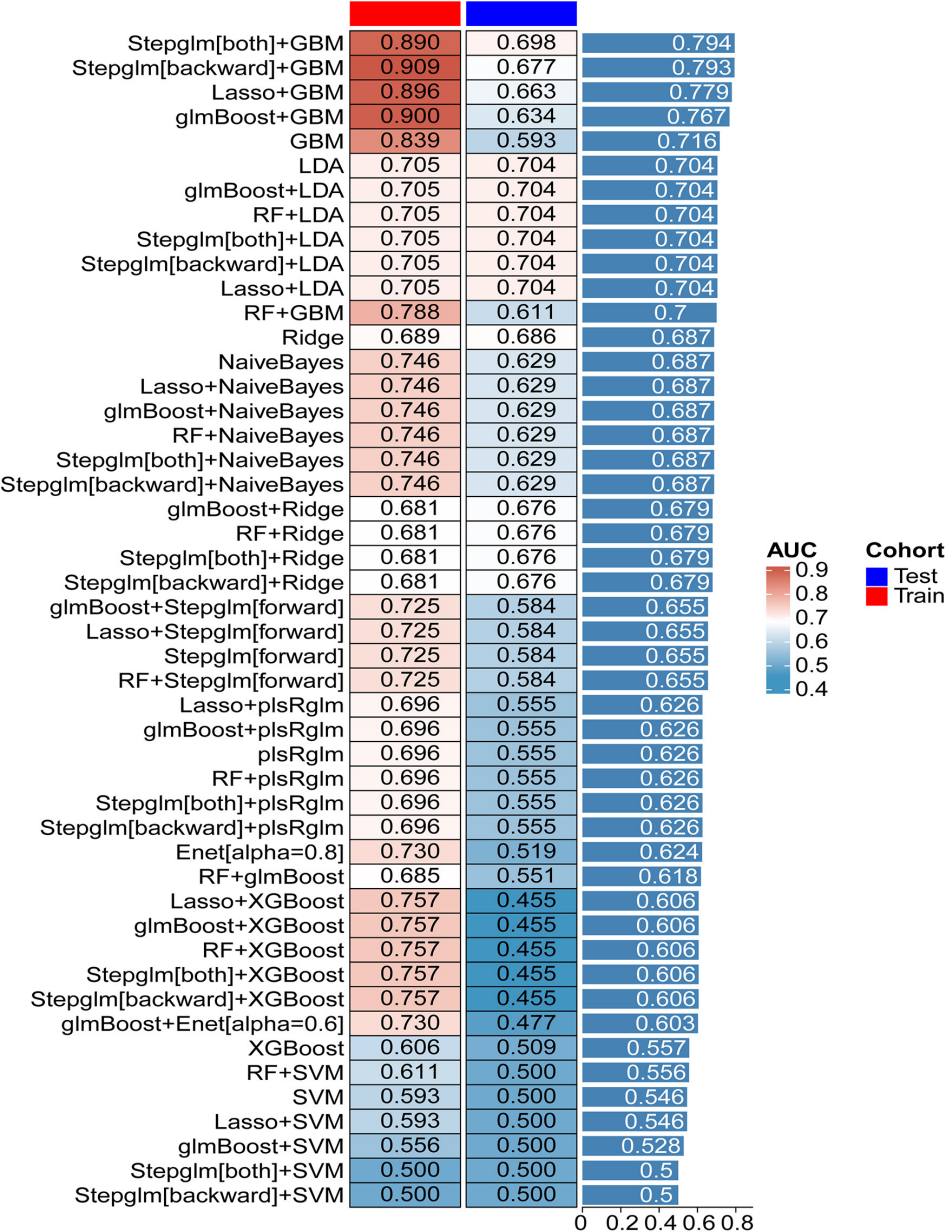
The performance of 101 predictive models in training and testing cohorts.

Twelve machine learning algorithms formed 113 combinations, with the data randomly split into a 7:3 ratio between the training (27 in the experiment group, 353 in the control group) and testing groups (11 in the experiment group, 151 in the control group). Figure 4 showcases the AUC values for 48 machine learning algorithms across the training, testing, and average groups. We observed significant differences in predictive performance among various models, not only demonstrating the tremendous potential of machine learning in clinical applications but also highlighting its limitations under certain circumstances. The “Stepglm[both] + GBM” model achieved an average AUC of 0.890 in the training set, indicating high accuracy in identifying mortality outcomes. This result suggests that the model can effectively integrate and analyze multiple clinical features, thus providing strong support for clinical practice. However, the model's AUC dropped to 0.794 during validation with the testing set. This phenomenon suggests that despite the model's excellent training performance, it harbors a risk of over-fitting when confronted with unknown data. This finding emphasizes the crucial importance of optimizing a model's generalization ability in machine learning applications to ensure its stability in real clinical settings. In comparison, the “Lasso + GBM” and “glmBoost + GBM” models also performed admirably, with AUCs of 0.896 and 0.779, and 0.900 and 0.767 in the training and testing sets, respectively. These results indicate that the models can achieve considerable efficiency in information extraction, especially when integrating significant biomarkers, to recognize and predict key clinical indicators. This allows clinicians to quantitatively assess patient risk and adjust corresponding treatment strategies based on these indicators. Particularly in the management of patients, timely warning and intervention can have a profound impact on survival rates. Additionally, in evaluating model performance, we found that some models, such as “RF + LDA” and “Ridge”, demonstrated relatively mediocre overall performance, with training set AUCs between 0.689 and 0.705, and testing set performances failing to exceed 0.704. This result suggests the need to pay attention to the gap between the training and testing sets, as well as the stability and applicability of the model during the selection process. The instability of a model may stem from various factors, including inadequacies in feature selection and sample size limitations.

We extracted the final selected variables for each machine learning model (Table 6). From the feature variable analysis, the indicators we adopted, such as white blood cell count, blood glucose, Temperature (body), lactate, and cholesterol, are critical physiological parameters directly related to the severity of the patient's condition. An elevated white blood cell count typically indicates severe infection, while high lactate levels suggest potential tissue hypoxia or shock. Changes in these physiological indicators can provide clinicians with important clues about the patient's physiological state. Furthermore, fluctuations in glucose and cholesterol levels may also reflect the patient's metabolic status, thereby having a significant impact on clinical management. Therefore, the construction of the model relies not only on the merits of the algorithms but also on a deep understanding and effective utilization of clinical data.

**Table 6:** The performance of 113 predictive models in training and testing cohorts.

Model	Train (Control: 353) (Experimental:27)	Test (Control: 152) (Experimental:11)	Average AUC	Features
Stepglm[both] + GBM	**0.890**	**0.698**	0.794	WBC Count/Glucose/Neutrophils Count/Cystatin C/Temperature/Sodium, Whole Blood/Cholesterol, Total/Absolute Lymphocyte Count/Urea/Lactate/Bilirubin, Total
Lasso + GBM	0.896	0.663	0.779	Glucose/Cholesterol, Total/Cystatin C/Absolute Lymphocyte Count/WBC Count/Bilirubin, Total/Chloride, Whole Blood/Albumin/Urea/Lactate/Neutrophils Count/Neutrophils/Temperature/Sodium, Whole Blood
glmBoost + GBM	0.900	0.634	0.767	Cholesterol, Total/Glucose/Absolute Lymphocyte Count/WBC Count/Cystatin C/Chloride, Whole Blood/Urea/Albumin/Lactate/Bilirubin, Total/Neutrophils Count/Neutrophils/Temperature/Sodium, Whole Blood
GBM	0.839	0.593	0.716	Glucose/Cholesterol, Total/WBC Count/Chloride, Whole Blood/Absolute Lymphocyte Count/Cystatin C/Albumin/Neutrophils Count/Sodium, Whole Blood/Urea/Bilirubin, Total/Lactate
LDA	0.705	0.704	0.704	Bilirubin, Total/Urea/Neutrophils/Temperature/Neutrophils Count/WBC Count/Cystatin C/Lactate/Chloride, Whole Blood/Cholesterol, Total/Absolute Lymphocyte Count/Glucose/Sodium, Whole Blood/Albumin
glmBoost + LDA	0.705	0.704	0.704	Bilirubin, Total/Urea/Neutrophils/Temperature/Neutrophils Count/WBC Count/Cystatin C/Lactate/Chloride, Whole Blood/Cholesterol, Total/Absolute Lymphocyte Count/Glucose/Sodium, Whole Blood/Albumin
RF + LDA	0.705	0.704	0.704	Bilirubin, Total/Urea/Neutrophils/Temperature/Neutrophils Count/WBC Count/Cystatin C/Lactate/Chloride, Whole Blood/Cholesterol, Total/Absolute Lymphocyte Count/Glucose/Sodium, Whole Blood/Albumin
Stepglm[both] + LDA	0.705	0.704	0.704	Bilirubin, Total/Urea/Neutrophils/Temperature/Neutrophils Count/WBC Count/Cystatin C/Lactate/Chloride, Whole Blood/Cholesterol, Total/Absolute Lymphocyte Count/Glucose/Sodium, Whole Blood/Albumin
Stepglm[backward] + LDA	0.705	0.704	0.704	Bilirubin, Total/Urea/Neutrophils/Temperature/Neutrophils Count/WBC Count/Cystatin C/Lactate/Chloride, Whole Blood/Cholesterol, Total/Absolute Lymphocyte Count/Glucose/Sodium, Whole Blood/Albumin
Lasso + LDA	0.705	0.704	0.704	Bilirubin, Total/Urea/Neutrophils/Temperature/Neutrophils Count/WBC Count/Cystatin C/Lactate/Chloride, Whole Blood/Cholesterol, Total/Absolute Lymphocyte Count/Glucose/Sodium, Whole Blood/Albumin
RF + GBM	0.788	0.611	0.700	Cholesterol, Total/Glucose/WBC Count/Neutrophils/Absolute Lymphocyte Count/Lactate/Albumin/Urea
Ridge	0.689	0.686	0.687	Bilirubin, Total/Urea/Neutrophils/Temperature/Neutrophils Count/WBC Count/Cystatin C/Lactate/Chloride, Whole Blood/Cholesterol, Total/Absolute Lymphocyte Count/Glucose/Sodium, Whole Blood/Albumin
glmBoost + Ridge	0.681	0.676	0.679	Bilirubin, Total/Urea/Neutrophils/Temperature/Neutrophils Count/WBC Count/Cystatin C/Lactate/Chloride, Whole Blood/Cholesterol, Total/Absolute Lymphocyte Count/Glucose/Sodium, Whole Blood/Albumin
RF + Ridge	0.681	0.676	0.679	Bilirubin, Total/Urea/Neutrophils/Temperature/Neutrophils Count/WBC Count/Cystatin C/Lactate/Chloride, Whole Blood/Cholesterol, Total/Absolute Lymphocyte Count/Glucose/Sodium, Whole Blood/Albumin
Stepglm[both] + Ridge	0.681	0.676	0.679	Bilirubin, Total/Urea/Neutrophils/Temperature/Neutrophils Count/WBC Count/Cystatin C/Lactate/Chloride, Whole Blood/Cholesterol, Total/Absolute Lymphocyte Count/Glucose/Sodium, Whole Blood/Albumin
Stepglm[backward] + Ridge	0.681	0.676	0.679	Bilirubin, Total/Urea/Neutrophils/Temperature/Neutrophils Count/WBC Count/Cystatin C/Lactate/Chloride, Whole Blood/Cholesterol, Total/Absolute Lymphocyte Count/Glucose/Sodium, Whole Blood/Albumin
Stepglm[backward] + GBM	0.909	0.677	0.677	Cholesterol, Total/Glucose/Absolute Lymphocyte Count/WBC Count/Cystatin C/Chloride, Whole Blood/Urea/Albumin/Lactate/Bilirubin, Total/Neutrophils Count/Neutrophils/Temperature/Sodium, Whole Blood
glmBoost + Stepglm[forward]	0.725	0.584	0.655	Bilirubin, Total/Urea/Neutrophils/Temperature/Neutrophils Count/WBC Count/Cystatin C/Lactate/Chloride, Whole Blood/Cholesterol, Total/Absolute Lymphocyte Count/Glucose/Sodium, Whole Blood/Albumin
Lasso + Stepglm[forward]	0.725	0.584	0.655	Bilirubin, Total/Urea/Neutrophils/Temperature/Neutrophils Count/WBC Count/Cystatin C/Lactate/Chloride, Whole Blood/Cholesterol, Total/Absolute Lymphocyte Count/Glucose/Sodium, Whole Blood/Albumin
Stepglm[forward]	0.725	0.584	0.655	Bilirubin, Total/Urea/Neutrophils/Temperature/Neutrophils Count/WBC Count/Cystatin C/Lactate/Chloride, Whole Blood/Cholesterol, Total/Absolute Lymphocyte Count/Glucose/Sodium, Whole Blood/Albumin
RF + Stepglm[forward]	0.725	0.584	0.655	Bilirubin, Total/Urea/Neutrophils/Temperature/Neutrophils Count/WBC Count/Cystatin C/Lactate/Chloride, Whole Blood/Cholesterol, Total/Absolute Lymphocyte Count/Glucose/Sodium, Whole Blood/Albumin
Lasso + plsRglm	0.696	0.555	0.626	Bilirubin, Total/Urea/Neutrophils/Temperature/Neutrophils Count/WBC Count/Cystatin C/Lactate/Chloride, Whole Blood/Cholesterol, Total/Absolute Lymphocyte Count/Glucose/Sodium, Whole Blood/Albumin
glmBoost + plsRglm	0.696	0.555	0.626	Bilirubin, Total/Urea/Neutrophils/Temperature/Neutrophils Count/WBC Count/Cystatin C/Lactate/Chloride, Whole Blood/Cholesterol, Total/Absolute Lymphocyte Count/Glucose/Sodium, Whole Blood/Albumin
plsRglm	0.696	0.555	0.626	Bilirubin, Total/Urea/Neutrophils/Temperature/Neutrophils Count/WBC Count/Cystatin C/Lactate/Chloride, Whole Blood/Cholesterol, Total/Absolute Lymphocyte Count/Glucose/Sodium, Whole Blood/Albumin
RF + plsRglm	0.696	0.555	0.626	Bilirubin, Total/Urea/Neutrophils/Temperature/Neutrophils Count/WBC Count/Cystatin C/Lactate/Chloride, Whole Blood/Cholesterol, Total/Absolute Lymphocyte Count/Glucose/Sodium, Whole Blood/Albumin
Stepglm[both] + plsRglm	0.696	0.555	0.626	Bilirubin, Total/Urea/Neutrophils/Temperature/Neutrophils Count/WBC Count/Cystatin C/Lactate/Chloride, Whole Blood/Cholesterol, Total/Absolute Lymphocyte Count/Glucose/Sodium, Whole Blood/Albumin
Stepglm[backward] + plsRglm	0.696	0.555	0.626	Bilirubin, Total/Urea/Neutrophils/Temperature/Neutrophils Count/WBC Count/Cystatin C/Lactate/Chloride, Whole Blood/Cholesterol, Total/Absolute Lymphocyte Count/Glucose/Sodium, Whole Blood/Albumin
Enet[alpha = 0.8]	0.730	0.519	0.624	Neutrophils/Temperature/Neutrophils Count/WBC Count/Lactate/Chloride, Whole Blood/Cholesterol, Total/Absolute Lymphocyte Count/Glucose/Sodium, Whole Blood/Albumin
RF + glmBoost	0.685	0.551	0.618	Neutrophils/WBC Count/Chloride, Whole Blood/Cholesterol, Total/Absolute Lymphocyte Count/Glucose/Sodium, Whole Blood
Lasso + XGBoost	0.757	0.455	0.606	Bilirubin, Total/Urea/Neutrophils/Temperature/Neutrophils Count/WBC Count/Cystatin C/Lactate/Chloride, Whole Blood/Cholesterol, Total/Absolute Lymphocyte Count/Glucose/Sodium, Whole Blood/Albumin
glmBoost + XGBoost	0.757	0.455	0.606	Bilirubin, Total/Urea/Neutrophils/Temperature/Neutrophils Count/WBC Count/Cystatin C/Lactate/Chloride, Whole Blood/Cholesterol, Total/Absolute Lymphocyte Count/Glucose/Sodium, Whole Blood/Albumin
RF + XGBoost	0.757	0.455	0.606	Bilirubin, Total/Urea/Neutrophils/Temperature/Neutrophils Count/WBC Count/Cystatin C/Lactate/Chloride, Whole Blood/Cholesterol, Total/Absolute Lymphocyte Count/Glucose/Sodium, Whole Blood/Albumin
Stepglm[both] + XGBoost	0.757	0.455	0.606	Bilirubin, Total/Urea/Neutrophils/Temperature/Neutrophils Count/WBC Count/Cystatin C/Lactate/Chloride, Whole Blood/Cholesterol, Total/Absolute Lymphocyte Count/Glucose/Sodium, Whole Blood/Albumin
Stepglm[backward] + XGBoost	0.757	0.455	0.606	Bilirubin, Total/Urea/Neutrophils/Temperature/Neutrophils Count/WBC Count/Cystatin C/Lactate/Chloride, Whole Blood/Cholesterol, Total/Absolute Lymphocyte Count/Glucose/Sodium, Whole Blood/Albumin
glmBoost + Enet[alpha = 0.6]	0.730	0.477	0.603	Neutrophils/Temperature/Neutrophils Count/WBC Count/Lactate/Chloride, Whole Blood/Cholesterol, Total/Absolute Lymphocyte Count/Glucose/Sodium, Whole Blood/Albumin
XGBoost	0.606	0.509	0.557	Bilirubin, Total/Urea/Neutrophils/Temperature/Neutrophils Count/WBC Count/Cystatin C/Lactate/Chloride, Whole Blood/Cholesterol, Total/Absolute Lymphocyte Count/Glucose/Sodium, Whole Blood/Albumin
RF + SVM	0.611	0.500	0.556	Bilirubin, Total/Urea/Neutrophils/Temperature/Neutrophils Count/WBC Count/Cystatin C/Lactate/Chloride, Whole Blood/Cholesterol, Total/Absolute Lymphocyte Count/Glucose/Sodium, Whole Blood/Albumin
SVM	0.593	0.500	0.546	Bilirubin, Total/Urea/Neutrophils/Temperature/Neutrophils Count/WBC Count/Cystatin C/Lactate/Chloride, Whole Blood/Cholesterol, Total/Absolute Lymphocyte Count/Glucose/Sodium, Whole Blood/Albumin
Lasso + SVM	0.593	0.500	0.546	Bilirubin, Total/Urea/Neutrophils/Temperature/Neutrophils Count/WBC Count/Cystatin C/Lactate/Chloride, Whole Blood/Cholesterol, Total/Absolute Lymphocyte Count/Glucose/Sodium, Whole Blood/Albumin
glmBoost + SVM	0.556	0.500	0.528	Bilirubin, Total/Urea/Neutrophils/Temperature/Neutrophils Count/WBC Count/Cystatin C/Lactate/Chloride, Whole Blood/Cholesterol, Total/Absolute Lymphocyte Count/Glucose/Sodium, Whole Blood/Albumin
Stepglm[both] + SVM	0.500	0.500	0.500	Bilirubin, Total/Urea/Neutrophils/Temperature/Neutrophils Count/WBC Count/Cystatin C/Lactate/Chloride, Whole Blood/Cholesterol, Total/Absolute Lymphocyte Count/Glucose/Sodium, Whole Blood/Albumin
Stepglm[backward] + SVM	0.500	0.500	0.500	Bilirubin, Total/Urea/Neutrophils/Temperature/Neutrophils Count/WBC Count/Cystatin C/Lactate/Chloride, Whole Blood/Cholesterol, Total/Absolute Lymphocyte Count/Glucose/Sodium, Whole Blood/Albumin

Bold values indicate statistical significance (*p* < 0.05).

## 4 Discussion

The objective of developing clinical predictive models is to facilitate risk stratification of outcomes for patients, thereby assisting clinicians and healthcare professionals in obtaining a comprehensive understanding of the patients' conditions. This, in turn, aids in making informed clinical decisions that can enhance the prognosis and quality of care for patients (34). In this study, we found that the combination of stepglm [both] and GBM produced the highest mean AUC curve, reflecting its superior performance in clinical prediction models. The stepglm [both] approach, which integrates both forward and backward selection processes, systematically identifies significant predictors while mitigating the effects of multicollinearity. This attribute is crucial in clinical contexts, where datasets often contain highly correlated variables. By narrowing down the predictor set to those with the most explanatory power, stepglm [both] creates a more parsimonious model, reducing noise and improving interpretability.

In contrast, algorithms such as Lasso and Ridge, while effective in regularization, may not fully leverage interactions among predictors due to their linear nature. This limitation can hinder their performance in capturing the complex relationships inherent in clinical data. GBM excels in modeling these complexities through its ensemble learning framework, which constructs multiple decision trees sequentially. Each tree learns from the errors of its predecessor, allowing the model to capture intricate patterns and interactions that linear models might overlook. Other methods, such as Naive Bayes and LDA, often rely on strong independence assumptions among predictors or require specific distributional assumptions that can be unrealistic in clinical settings. Consequently, these algorithms may struggle to generalize well, particularly with diverse patient populations or multifaceted clinical scenarios. Furthermore, while tree-based methods like Random Forest also provide flexibility and robustness, they can sometimes suffer from over-fitting, especially in high-dimensional spaces. GBM, with its advanced techniques for controlling over-fitting, demonstrates superior adaptability and accuracy in this regard. In conclusion, The synergy of stepglm [both] with GBM results in a robust model that not only selects pertinent features but also intelligently combines them to optimize predictive power. This highlights the necessity of employing advanced, integrated approaches in developing predictive models, particularly in the context of sophisticated clinical datasets. Future research should continue to explore such hybrid models to further enhance prediction accuracy and support clinical decision-making.

This study identified a set of 12 variables, including WBC Count, Glucose, Neutrophils Count, Cystatin C, Temperature (body), Sodium (Whole Blood), Cholesterol (Total), Absolute Lymphocyte Count, Urea, Lactate, and Bilirubin (Total). However, we recently noticed a study indicating that white blood cell (WBC) count, neutrophil count (NEU), eosinophil count (EO), and hemoglobin (HGB) levels are significantly associated with an increased risk of pediatric pneumonia (35). By carefully comparing our results with this study, we found that WBC and NEU are commonly shared variables, which exhibit significant prognostic value in our study. Additionally, Lu's article focuses on the association between prenatal and perinatal exposure to industrial air pollutants and childhood pneumonia, exploring potential mechanisms through blood biomarkers (35).Our prognostic model supports diagnosis, treatment, and early intervention, covering a broader range of indicators such as inflammatory markers (WBC, neutrophils), metabolic indicators (blood glucose, total cholesterol, urea), and organ function indicators (cystatin C, lactic acid). Lu's work through environmental epidemiology has revealed the long-term health effects of SO₂ exposure, emphasizing the importance of preventive medicine (35). Our research, on the other hand, has identified multi-dimensional prognostic variables from clinical data to support precision medicine. Certainly, in future studies, we aim to incorporate environmental exposure data such as SO₂ concentration into our prognostic models, enabling comprehensive assessments of both individual biological states and external environmental risks, achieving a holistic evaluation of the “individual-environment” interaction.

Furthermore, researchers have found that the WBC count, being an easily obtainable biomarker, can aid in identifying CAP patients who are more likely to benefit from adjunctive dexamethasone therapy (36). When used in conjunction with blood heparin-binding protein and the neutrophil-to-lymphocyte ratio, it can improve diagnostic specificity for critically ill patients (84.13%) (37). Our study is consistent with this evidence, although cohort analyses indicate that the WBC count may not be helpful in predicting non-severe and severe diseases in pediatric patients (38). We believe this discrepancy might be due to population differences across countries or the WBC count being a non-independent prognostic factor. Findings indicate that PFKFB3 is a molecular switch that regulates the use of glucose vs. fructose in glycolysis, thereby enhancing our understanding of lung endothelial cell metabolism during respiratory failure (39). Reducing neutrophil count has been shown to mitigate lung injury in murine models of pneumococcal pneumonia (40) and is significantly negatively correlated with respiratory failure in COVID-19 patients (41). Reviewing cohort analyses confirms that plasma Cystatin C levels decrease in CAP patients following antibiotic treatment (42). Additionally, serum Cystatin C within 24 h of admission appears to be a marker for predicting acute kidney injury in CAP patients (43). An observational study found that patients with elevated temperatures within the first 48 h of ICU admission had higher survival rates (44). Retrospective analysis revealed that hyponatremia not only increases morbidity in CAP patients but is also an independent predictor of prolonged hospitalization (45).

Cholesterol has been identified as a risk factor for CAP in young South Korean soldiers, with the case group's levels lower than those of the control group (46). Inhibiting cholesterol ester transfer protein has even been shown to reduce mortality in murine models of pneumococcal-induced sepsis (47). Studies indicate that the absolute lymphocyte count at the time of pneumonia diagnosis can serve as a prognostic factor for postoperative pneumonia patients (48). The absolute lymphocyte count at admission can even distinguish between SARS and CAP cases (49). A single-center retrospective cohort study found that a higher Urea-to-Albumin Ratio at the onset of ICU admission is independently associated with increased in-hospital mortality in patients with severe pneumonia (50). The criteria encompassing Confusion, Urea, Respiratory Rate, and Shock Index or Adjusted Shock Index predict mortality in community-acquired pneumonia (51). It has been reported that patients who die, are hospitalized, or admitted to the ICU due to pneumonia exhibit higher lactate levels (52). Initial blood lactate was an independent outcome predictor in COVID-19 ICU patients (53). A significant decrease in Bilirubin, Total levels was observed among survivors of severe pneumonia (54), while an increase was significantly more prominent in the mortality group of COVID patients compared to the survivors (55). Children with severe pneumonia displayed elevated levels of Bilirubin, Total, and uric acid (56).

Reviewing these findings reveals that the 12 variables mentioned are associated with the severity and prognosis of pneumonia, although there have been no reports of these variables collectively serving as prognostic factors. The relevance of these predictors in clinical practice reflects the physiological state and disease severity of patients. The construction of predictive models demonstrates the potential of machine learning technology in clinical applications. This outcome not only underscores the importance of comprehensive biochemical markers in prognosis assessment but also provides a robust foundation for clinical decision-making. In practice, early identification and intervention based on these markers enable clinicians to formulate more effective personalized treatment plans, thereby improving patient outcomes. Moreover, the study's results offer valuable insights for similar applications in the management of other diseases, further propelling the advancement of precision medicine. Consequently, this finding holds significant clinical implications and opens new avenues for research in the field of pediatric critical care.

## 5 Conclusions

In this study, we employed a variety of statistical methods to identify laboratory test parameters associated with the prognosis of pediatric pneumonia patients, identifying 25 test indicators closely related to ICU mortality. Linear regression analysis revealed a linear correlation between 11 variables and outcomes, while logistic regression elucidated the influence of 11 indicators. A stepwise regression algorithm preliminarily selected 28 variables from the original 78 for subsequent analysis, and the intersection of the Top 20 correlated features from Spearman and Pearson algorithms yielded 14 model construction factors. We obtained various variable interaction coefficients through Random Forest (RF) and Grey Relational Analysis. Among 113 machine learning algorithm combinations, “Stepglm [both] + GBM” exhibited the highest prognostic accuracy, with 89% accuracy in the training set, an AUC of 0.698 in the test set, and an average AUC of 0.794.

In this study, we aimed to identify laboratory test parameters that are associated with the prognosis of pediatric pneumonia patients. While our findings are promising, their translation into clinical practice requires further exploration and collaboration with clinicians. To facilitate the application of our research findings in clinical decision-making, we plan to develop clinical decision support tools in collaboration with clinicians. Naturally, our research has several limitations. Due to data scarcity, we could only distinguish between the training and test sets within the cohort, lacking external cohort validation. The impact of data scarcity and the relatively small sample size (only 543 cases after data preprocessing) on the research conclusions cannot be overstated. Given the current trend in medical big data, our sample size is insufficient for a comprehensive assessment of the disease status. To quantify the possible deviation range and its potential impact on variable screening and model accuracy, further studies with larger sample sizes are warranted. To address these limitations and construct a mature clinical prediction model, continuous expansion of the sample size and optimization of algorithm parameters are necessary. In the future, based on this study, we plan to design prospective cohort studies to establish our own database. Additionally, we aim to conduct in-depth research on the specific impact mechanisms of these test indicators on pediatric pneumonia survival rates. By understanding the underlying mechanisms, we can enhance the prognosis and survival rates of patients.

Moreover, we recognize the need for interdisciplinary collaboration. Integrating our research findings into web applications or programs can facilitate clinicians and scholars in predicting disease trends, enabling early preventive measures and interventions. Collaborations with clinicians in further developing our research into clinical decision support tools or processes would be beneficial for the practical application of our findings. In summary, while our study provides valuable insights, its translation into clinical practice requires further collaborations and validations, particularly with regard to sample size expansion and interdisciplinary collaborations.

## Data Availability

The original contributions presented in the study are included in the article/Supplementary Material, further inquiries can be directed to the corresponding author.
